# Significant Type I and Type III Collagen Production from Human Periodontal Ligament Fibroblasts in 3D Peptide Scaffolds without Extra Growth Factors

**DOI:** 10.1371/journal.pone.0010305

**Published:** 2010-04-22

**Authors:** Yoshiyuki Kumada, Shuguang Zhang

**Affiliations:** 1 Center for Biomedical Engineering, Massachusetts Institute of Technology, Cambridge, Massachusetts, United States of America; 2 Olympus America Inc., Center Valley, Pennsylvania, United States of America; Illinois Institute of Technology, United States of America

## Abstract

We here report the development of two peptide scaffolds designed for periodontal ligament fibroblasts. The scaffolds consist of one of the pure self-assembling peptide scaffolds RADA16 through direct coupling to short biologically active motifs. The motifs are 2-unit RGD binding sequence PRG (PRGDSGYRGDS) and laminin cell adhesion motif PDS (PDSGR). RGD and laminin have been previously shown to promote specific biological activities including periodontal ligament fibroblasts adhesion, proliferation and protein production. Compared to the pure RADA16 peptide scaffold, we here show that these designer peptide scaffolds significantly promote human periodontal ligament fibroblasts to proliferate and migrate into the scaffolds (for ∼300 µm/two weeks). Moreover these peptide scaffolds significantly stimulated periodontal ligament fibroblasts to produce extracellular matrix proteins without using extra additional growth factors. Immunofluorescent images clearly demonstrated that the peptide scaffolds were almost completely covered with type I and type III collagens which were main protein components of periodontal ligament. Our results suggest that these designer self-assembling peptide nanofiber scaffolds may be useful for promoting wound healing and especially periodontal ligament tissue regeneration.

## Introduction

It has previously been reported that a class of designer self-assembling peptide scaffolds have wide application including for 3-D cell culture, drug delivery, regenerative medicine and tissue engineering [1]–[5]. The class of self-assembling peptide materials can undergo spontaneous assembly into well-ordered nanofibers and scaffolds, ∼10nm in fiber diameter with pores between 5–200nm and over 90% water content [6]. These peptide scaffolds have 3-D nanofiber structures similar to the natural extracelluar matrix including collagen. And the scaffolds are biodegradable by a variety of proteases in a body with superior biocompatibility with tissue [7]. Moreover, these scaffolds can be modified and functionalized by direct extension of peptides with known biologically functional peptide motifs to promote specific cellular responses. One family of these peptide scaffolds, functionalized RADA16 (AcN-RADARADARADARADA-CONH_2_) has been studied for bone, cartilage, neural regeneration and angiogenesis promotion [8]–[11].

In treatment of periodontal disease, a number of surgical techniques have been developed to regenerate periodontal tissue, including guided tissue regeneration [12]–[14], bone grafting [15], [16], enamel matrix derivative [17]–[19] and the use of growth factors [20]–[25]. However, these animal-derived biomaterials have recently become several concerns for clinical use including the risk of infection agents from animals to human and the difficulty of handling. Also, the efficacy of these animal-derived biomaterials on periodontal ligament fibroblasts still remains unknown [7]. A product using human recombinant growth factor is currently commercially available for treatment of periodontal disease. Platelet-derived growth factor PDGF has been reported to promote periodontal ligament fibroblasts proliferation and proteins synthesis including collagen [26]. But the growth factors are expensive, and many doses may be required to achieve any therapeutic effect. Therefore, a simple, safe and inexpensive method for periodontal tissue regeneration is required.

Recently, several peptide molecules including RGD and laminin cell adhesion motifs have been reported to promote periodontal ligament fibroblasts activities [27]–[29]. RGD (Arg-Gly-Asp) is well known as a key binding sequence for cell attachment specifically working with integrin. Laminin is a main component of basement membrane. The basement membrane is not only important as a structural component supporting cell, but also gives to the cells an instructive microenvironment that modulates their function. Cell adhesion is a first phase of cell/material interaction and influences the cell's capacity to proliferate, migrate and differentiation. Therefore the fully-synthesis peptide scaffolds functionalized by RGD and laminin cell adhesion motifs show promise as a simple, safe and inexpensive material for periodontal therapy.

We here studied periodontal ligament fibroblasts activities on two designer self-assembling peptide scaffolds PRG and PDS in vitro. PRG is peptide scaffold RADA16 through direct coupling to a 2-unit RGD binding sequence PRGDSGYRGDS. PDS is RADA16 through direct coupling to a laminin cell adhesion motif PDSGR. And these scaffolds significantly promote periodontal ligament fibroblasts cell attachment, proliferation, migration and extracellular matrix proteins production, especially type I and type III collagens, which are major extracellular matrix protein components of periodontal ligament [30], [31]. These results suggest that the designer peptide scaffolds may be useful for wound care and especially periodontal tissue regeneration.

## Results

### Synthesis of new designer self-assembling peptides

**Figure 1:**
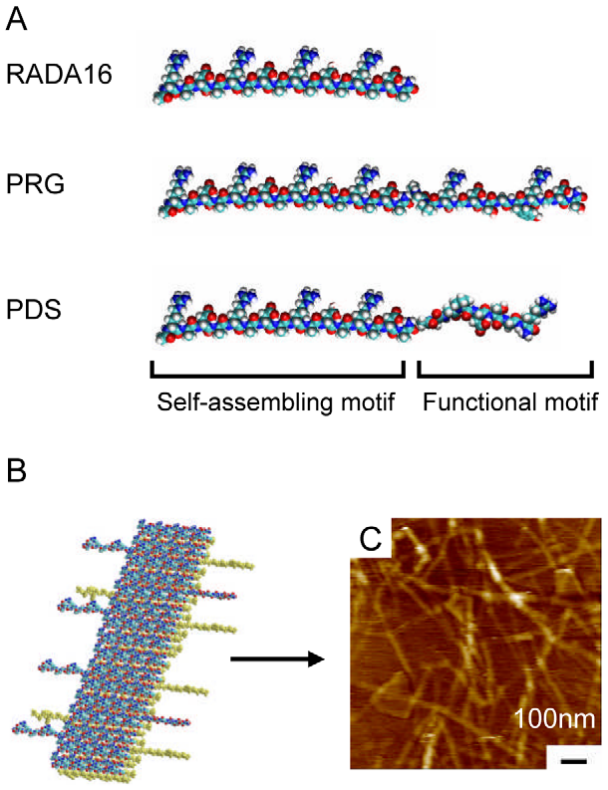
Molecular model of designer peptides and nanofiber. A) Molecular models of designer peptides RADA16, PRG and PDS. B) Molecular model of self-assembling peptide nanofibers formation with PRG peptide, representing a beta-sheet structure. Note the sequences PRG extending out from the nanofiber. C) Typical AFM morphology of a self-assembling peptide nanofiber scaffold PRG mixed with RADA16. (Photograph by Akihiro Horii).

The designer self-assembling peptide RADA16 was functionalized with RGD and laminin cell adhesion motifs in order to develop mimic extracellular matrix that enhance periodontal ligament fibroblasts maintenance and function in vitro. These designer peptides PRG and PDS were synthesized by direct extension from the C-terminal of the self-assembling peptide RADA16 using solid phase synthesis with different functional peptide motifs (Fig. 1 A). They were 2 units of RGD sequence (PRGDSGYRGDS) and cell adhesion motif of laminin (PDSGR). Glycine residues were used between the self-assembling motif (RADA)_4_ and the functional motif as a space linker for keeping the flexibility of functional peptides. These designer peptide sequences are listed in Table 1.

**Table 1:** Designer self-assembling peptides used in this study.

Name	Sequences	Description
RADA16	Ac-(RADA)_4_-CONH_2_	Designer self-assembling motif
PRG	Ac-(RADA)_4_-GPRGDSGYRGDS-CONH_2_	2-unit RGD motifs
PDS	Ac-(RADA)_4_-GGPDSGR-CONH_2_	From laminin cell adhesion domain (PDSGR)

The sequences are from N→C. Ac  =  acetylated N-termini, -CONH_2_  =  amidated C-termini. The peptide motif souces from various protein origins.

The peptides were solubilized in water at a concentration of 10mg/ml (1%, w/v). The pure designer peptides PRG and PDS undergo self-assembling to form soft hydrogels. Mixing with self-assembling peptide RADA16 facilitated the self-assembling and gelation. It has previously been reported that a molecular models of β-sheet structure of functionalized peptides were proposed and fiber structures of functionalized peptide scaffolds were observed by AFM (Atomic force microscopy) experiments [9], [10]. A molecular model representing the self-assembling peptide nanofiber with PRG are shown in Fig. 1 B. Typical AFM image in Fig. 1 C shows nanofiber formation of the peptides [10].

### Periodontal ligament fibroblasts attachment

**Figure 2:**
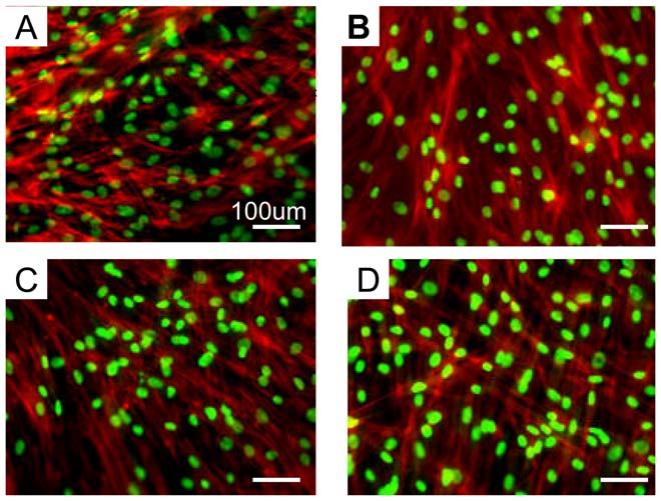
Cell morphology on the different scaffolds after two weeks culture. Fluorescence microscopy image of periodontal ligament fibroblasts A) on RADA16, B) on PRG, C) on PDS and D) on rat type I Collagen as a positive control. Fluorescenct staining with Rhodamin phalloidin for F-actin (red) and SYTOX Green for nuclei (green) showed the cell attachments and distributions. The scale bar represents 100um for all images.

In order to evaluate periodontal ligament fibroblasts attachment and growth on these peptide scaffolds, the same number of cells were seeded on the different scaffolds and cultured for two weeks. Rat type I collagen gel was used as a positive control. Fig. 2 A–D showed the typical morphologies of periodontal ligament fibroblasts on different scaffolds. Periodontal ligament fibroblasts adhered well to each scaffold and spread on the surface of the scaffold. Each scaffold was almost covered with periodontal ligament fibroblasts and monolayer formations were observed. Cell morphologies on peptide scaffolds RADA16, PRG and PDS were quite similar with that on rat type I collagen gel. These results suggest that the peptide scaffold surfaces present similar properties to the natural extracellular matrix in periodontal ligament fibroblasts attachment.

### Periodontal ligament fibroblasts growth on functionalized peptide scaffolds

**Figure 3:**
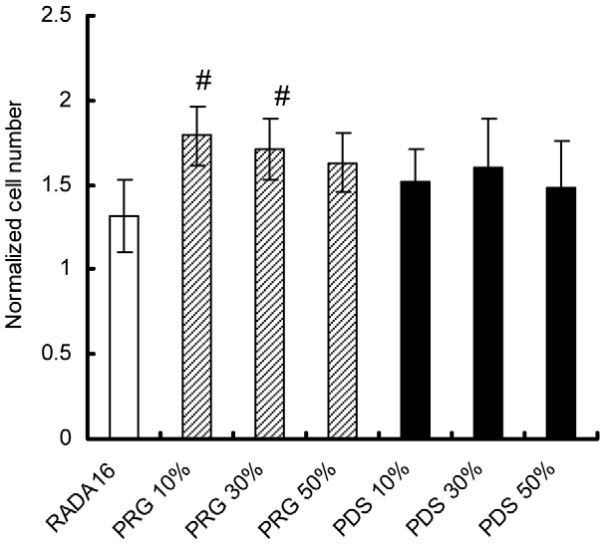
Cell densities on the different scaffolds of different mix ratio of designer PRG/PDS and pure RADA16 after two weeks culture. Initial seeding density (255 cells/mm^2^) was used to calculate fold changes in cell densities after two weeks in culture for each of the scaffolds. There is a tendency of periodontal ligament fibroblasts to proliferate on functionalized peptide scaffolds PRG and PDS. The fibroblasts proliferated significantly on PRG 10% and 30% compared to RADA16 (#ρ<0.01 vs RADA16). PRG/PDS concentration in the mix as low as 10–30% seems to be effective for growing the fibroblasts.

The growth of periodontal ligament fibroblasts on each scaffold was analyzed in Fig. 3. After the 2-week culture, cell density was calculated for each condition. Interestingly, cell densities increased on functionalized peptide scaffolds PRG and PDS with respect to RADA16. They suggest that the cell adhesion motifs RGD and PDSGR of the functionalized peptide scaffolds increased the number of initially attached fibroblasts and accelerated the fibroblasts proliferation on the scaffolds. We also analyzed the effects of mix ratio of designer peptide and pure peptide. Periodontal ligament fibroblasts were cultured on the different scaffolds consisting of a different mix ratio (10%, 30% and 50%) of designer peptides PRG/PDS and pure peptide RADA16. The result shows that PRG/PDS concentration in the mix as low as 10–30% seems to be effective for growing the fibroblasts.

### Periodontal ligament fibroblasts migration into functionalized peptide scaffolds

**Figure 4:**
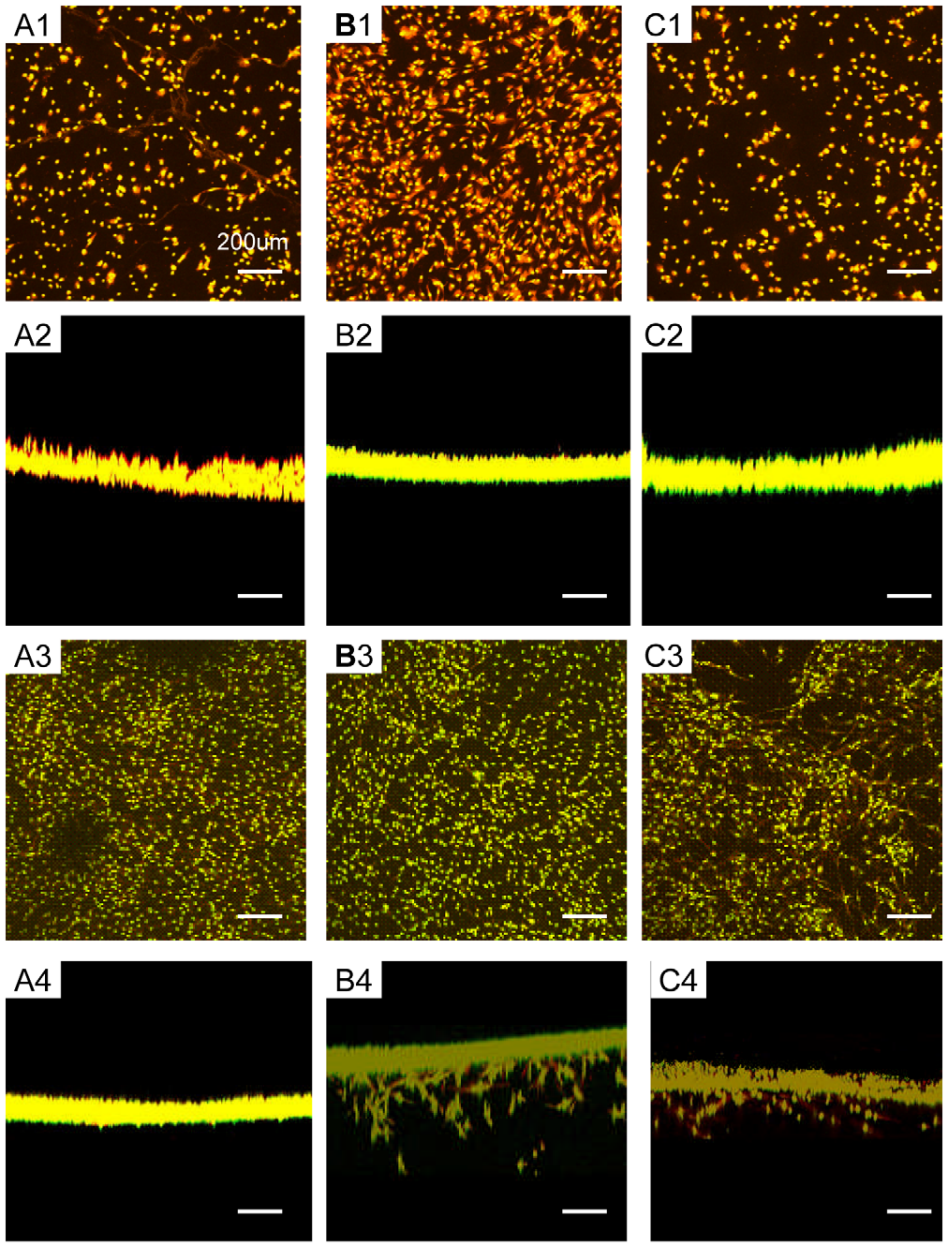
Constructed images of 3-D confocal microscopy images of periodontal ligament fibroblasts on the different scaffolds. Fluorescent staining with Rhodamin phalloidin and SYTOX Green. A) RADA16, B) PRG and C) PDS. A1, B1,C1) Vertical and A2, B2,C2) horizontal images after five hours culture. A3, B3, C3) Vertical and A4, B4, C4) horizontal images after two weeks culture. There were significant cell migrations into the functionalized peptide scaffolds PRG and PDS after two weeks. The scale bar represents 200 um for all images.

Spontaneous cell migrations were observed with the confocal microscopy 3-D image collections and reconstructions. These results in Fig. 4 showed the reconstruction images of periodontal ligament fibroblasts in RADA16 (A1–4), PRG (B1–4) and PDS (C1–4). The migrated cells could be clearly visualized with the confocal imaging and the difference of the characteristics in these scaffolds was found. Periodontal ligament fibroblasts spread well on the surface of the scaffold RADA16 but didn't migrate into the scaffold at all (A4). On the other hand, the fibroblasts on functionalized peptide scaffolds PRG and PDS not only spread well on the surface, but also spontaneously migrated into the scaffolds ∼300 µm (B4, C4).

### Type I and type III collagen productions from periodontal ligament fibroblasts in functionalized peptide scaffolds

**Figure 5:**
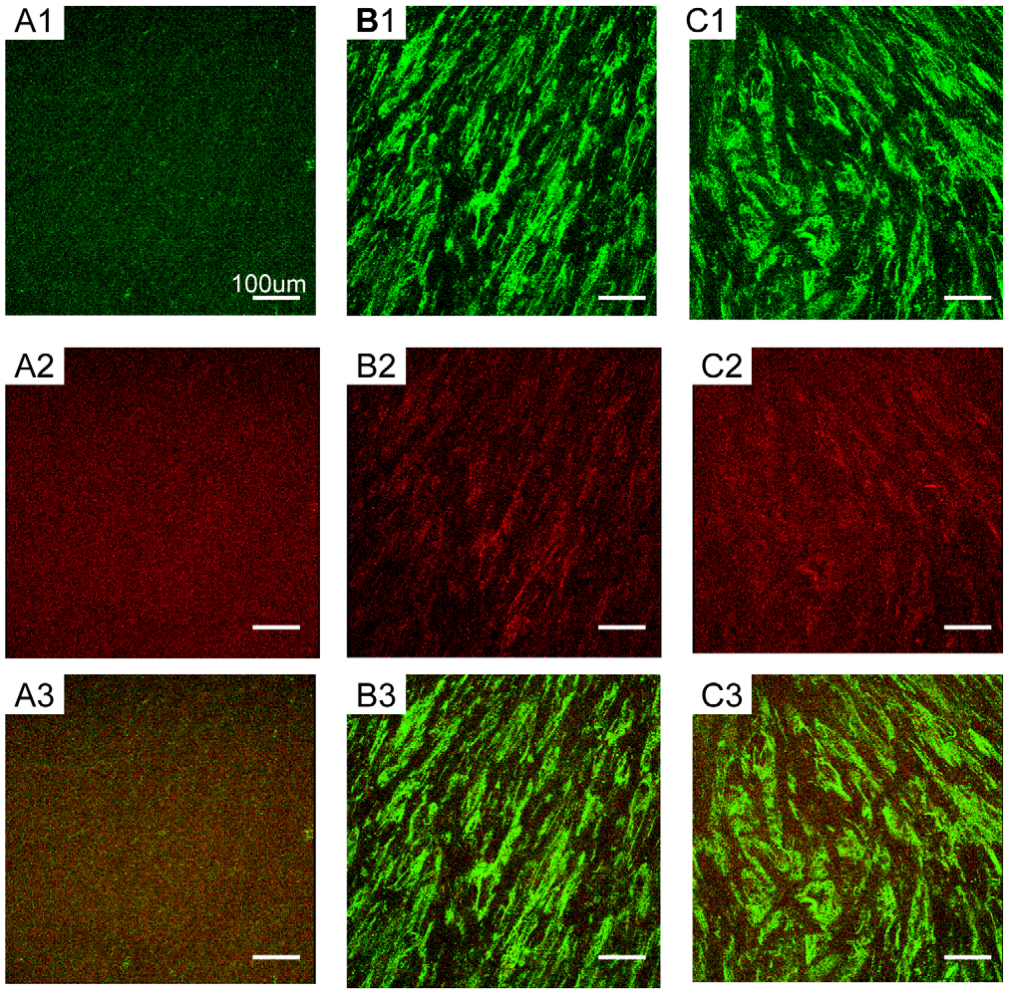
Type I and type III Collagens fluorescent immunostaining images of periodontal ligament fibroblasts on the different scaffolds after six weeks culture. Fluorescent immunostaining with Anti-collagen type I and Alexa fluor 488 goat anti-rabbit IgG for collagen type I (green) in A1) RADA16, B1) PRG and C1) PDS, and Anti-collagen type III and Alexa fluor 594 goat anti-mouse IgG for collagen type III (red) in A2) RADA16, B2) PRG and C2) PDS. Mix ratio of designer PRG/PDS and RADA16 scaffold is 1∶9. In case of PRG and PDS, periodontal ligament fibroblasts drastically produced type I and type III collagens which were extra-cellular matrix components of periodontal ligament. The scale bar represents 100 um for all images.

In order to evaluate an extracellular matrix proteins production from periodontal ligament fibroblasts, we performed fluorescent immunostaining technique to visualize type I and type III collagens in the peptide scaffolds with confocal microscope. Type I and type III collagens were well known as major protein components of periodontal ligament, produced by periodontal ligament fibroblasts [30], [31]. Fig. 5 showed type I (green) and type III (red) collagen images in peptide scaffolds RADA16 (A), PRG (B) and PDS (C). These images were captured under the same viewing condition. Type I and type III collagens were clearly observed in functionalized peptide scaffolds PRG and PDS in comparison to RADA16. The collagens in functionalized peptides PRG and PDS seemed to be produced by the periodontal ligament fibroblasts and appeared along the fibroblast orientation. They almost covered the entire surface of the peptide scaffolds. Type III collagen seemed to be less than type I collagen. This result is consistent with the structure of periodontal ligament [30]. On the other hand, collagens weren't clearly observed in RADA16. According to Fig. 2 and 4, periodontal ligament fibroblasts remained confined to the surface of the scaffold RADA16 and didn't grow three dimensionally. These results suggest that these functionalized peptide scaffolds provide hospitable three-dimensional microenvironment for periodontal ligament fibroblasts to retain the characteristics and grow well as if the fibroblasts were in the natural periodontal ligament.

These are significant findings that a simple functional motif could have drastic influence on cell behaviors, especially cell migration and collagen productions. It is much easier and less expensive to produce the designer scaffold than to find complex and expensive soluble growth factors that show similar cell behavior.

## Discussion

**Figure 6:**
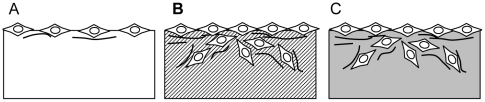
Schematic illustration of the results. A) Periodontal ligament fibroblasts on the peptide scaffold RADA16, B) on the functionalized peptide scaffold PRG and C) on the functionalized peptide scaffold PDS. In case of the functionalized peptide scaffold PRG and PDS, periodontal ligament fibroblasts showed cell proliferation, migration into the scaffolds and type I and type III collagen productions required to regenerate periodontal ligament.

In this work we described the development of self-assembling peptide scaffolds with similar properties to natural extracellular matrix proteins for periodontal tissue regeneration. We selected two sequence motifs from 2 unit RGD binding sequence PRGDSGYRGDS and laminin cell adhesion motif PDSGR. Fig. 6 summarized the results mentioned above. The two motifs seemed to be effective for periodontal ligament fibroblasts 3-D growth and collagens production from the fibroblast.

The designer peptide scaffold PRG contains RGD cell attachment motif for integrin receptors. It has been reported that PRG promoted osteoblast activity for bone tissue regeneration and endothelial cell activity for angiogenesis [10], [11]. Another designer peptide scaffold PDS contains PDSGR cell attachment motif of laminin. It has been reported that periodontal ligament fibroblasts adhered to RGD motif, fibronectin and laminin, and expressed the integrin subunits related to the attachment to these extracellular matrix proteins [27], [28]. In this study, we showed that PRG and PDS promoted periodontal ligament fibroblasts activity. This suggests that periodontal ligament fibroblasts indeed recognized the exposed adhesion motifs attached to each scaffold through the integrin receptors for RGD motif or cell attachment motif of laminin PDSGR. Periodontal ligament fibroblasts adhered to the surface of these peptides recognized the adhesion motif inside of the scaffold as well and then migrated into these scaffolds.

It is known that growth factors and mechanical signals regulate the production of extracellular matrix proteins of fibroblasts [26], [31]–[38]. Considering that no extra growth factors were added, our results suggest that the biochemical and perhaps mechanical signals from each different peptide scaffolds induced the production of extracellular matrix proteins. It has been previously reported that fibroblasts translate mechanical signals into changes in extracellular matrix production through the integrin [39]–[41]. They indicated that stretching of matrix - integrin contacts leads to cytoskeleton-mediated signals by rearrangement of cytoskeletal components that include actin filaments and recruitment of kinases such as focal adhesion kinase (FAK) and Src. Activation of FAK and Src further activate mitogen activated protein kinase (MARK) signaling pathway to promote gene transcription. The altered gene transcription leads to translational and post-translational modification to selectively synthesize and secrete extracellular matrix proteins. In our study, it would appear that the interaction between integrin of the fibroblasts – cell adhesion motifs of the scaffolds PRG and PDS triggers an intracellular signaling pathway described above, then the fibroblasts synthesize and secrete type I and III collagens. On the other hand, since peptide scaffold RADA16 does not have cell adhesion motif, periodontal ligament fibroblasts seem to adhere to peptide by a different way. It has been assumed that interaction between charged residues of RADA16 and cell surface components play a role in non-integrin-mediated cell attachment to the peptide scaffold [42], and cells adhere to the peptide scaffold via adhesion proteins which can be derived either from the serum of the added culture medium or produced by the cells [43]. Periodontal ligament fibroblasts seem to adhere to the peptide scaffold RADA16 without the interaction between integrin-cell adhesion motifs of scaffold and not to produce collagens as in the case of the functionalized peptide scaffolds PRG and PDS. These results suggest that peptide scaffold functionalization with cell adhesion motifs which interact with integrin may be useful to stimulate matrix protein production from fibroblasts.

The periodontium, the supporting teeth apparatus, consists of four tissues, gingival, periodontal ligament, cementum and alveolar bone. The diverse composition of the periodontium makes periodontal wound healing a complex process because of the interaction between hard and soft connective tissues, implying the selective repopulation of the root surface by cells capable of reforming the cellular and extracellular components of new periodontal ligament, cementum and alveolar bone [44]. Guided tissue regeneration is a conventional method for periodontal tissue reconstruction, which could be driven by excluding or restricting the repopulation of periodontal defects by epithelial and gingival connective cells, providing space and favorable niche to maximize periodontal ligament fibroblasts, cementoblasts and osteoblasts to migrate selectively, proliferate and differentiate. Considering the clinical use of these scaffolds for periodontal tissue reconstruction, they are required to provide the selective cell repopulations. It has been previously reported that the peptide scaffolds PRG could control osteoblasts activities by changing the concentration of the designer peptide containing two unit of RGD [10]. It also has been reported that laminin has specific cell adhesion properties, which periodontal ligament fibroblasts and osteoblasts could adhere well, compared with gingival fibroblasts [27]–[29]. They suggest that these peptide scaffolds PRG and PDS with laminin cell adhesion motif might be useful as periodontal tissue filler with selective cell repopulation properties.

We have developed and evaluated two designer functionalized self-assembling peptide scaffolds for periodontal ligament regeneration through directly coupling RADA16 with short biologically cell attachment motifs. In our study these designer functionalized peptide scaffolds PRG and PDS have been demonstrated to significantly enhance periodontal ligament fibroblasts proliferation, migration and extracellular matrix protein type I and type III collagen production in cell culture. Thus these designer scaffolds will be likely very useful to reconstruct periodontal tissue.

## Materials and Methods

### Peptide solution preparation and gel formation

RADA16 was purchased as PuraMatrix™ from BD Bioscience, Bedford, MA. The designer peptides PRG and PDS were custom-synthesized by CPC Scientific (Purity >80%, San Jose, CA). They were dissolved in water at a final concentration of 1% (w/v, 10mg/ml) and sonicated for 20 min.(aquasonic, model 50T, VWR, NJ). After sonication, they were filter-sterilized (Acrodisc Syringe Filter, 0.2µmHT Tuffrun membrane, Pall Corp., Ann Arbor, MI) for succeeding uses. The designer functionalized peptide solutions were mixed in a volume ratio of 1∶1 with 1% PuraMatrix solution, except otherwise stated. Desired number of cell culture inserts (10mm diameter, 0.4 µm pore size, BD Bioscience, Bedford, MA) were placed in a 24-well culture plate with 250µl culture medium in each well. 100µl peptide solution was loaded directly into each of the inserts and then incubated for at least 1 hour at 37°C for gelation. 100µl of culture medium were added onto the gel and then incubated overnight at 37°C. Once the gel was formed, the medium was removed and changed twice more to equilibrate the gel to physiological pH prior to plating the cells. Six samples for each scaffold were prepared.

### Collagen gel formation

Rat tail collagen type I was purchased from BD Bioscience (Bedford, MA) and prepared according to the manufacturer's protocols. The same volume (100µl) of collagen type I (2.5mg/mg) was plated on the cell culture inserts and allowed to gel for 30 minutes at 37°C, with subsequent addition of culture medium.

### Cell culture of periodontal ligament fibroblasts

Primary isolated human periodontal ligament fibroblasts were commercially obtained from Lonza Inc. (HPDLF, Walkersville, MD) and routinely grown in the culture medium (SCGM, Walkersville, MD) on regular cell culture flask. The cells were plated at 2×10^4^ cells on the gel in the inserts. The culture medium was changed every three days. No additional growth factors were used for all cultures.

### Fluorescence microscopy

Following the experiments, the cells on the gel were fixed with 4% paraformaldehyde for 15 min and permeabilized with 0.1% Triton X-100 for 5 min at room temperature. Fluorescent Rhodamin phalloidin and SYTOX® Green (Molecular Probes, Eugene, OR) were used for labeling F-actin and nuclei, respectively. Images were taken using a fluorescence microscope (Axiovert 25, ZEISS) or laser confocal scanning microscope (Olympus FV300). Cell numbers were counted in three random fields per substrate with the aid of the fluorescence microscope above with objective 10×. Cell densities were then calculated using these cell numbers counted and the magnifying power of the microscope.

### Fluorescent immunostaining for type I and type III collagens visualization

After the cell fixation, the primary antibody for type I collagen (5% Anti-collagen type I, Millpore, MA) was added and incubated at 37°C for 40 min, then washing six times with PBS with 1% BSA. The second antibody (0.5% Alexa fluor 488 goat anti-rabbit IgG, Invitrogen) was added and incubated at 37°C for another 40 min, then washing as well. After that, the primary antibody for type III collagen (0.5% Anti-collagen type III, Millpore, MA) was added and incubated at 37°C for 40 min, then washing. Finally, the second antibody (0.5% Alexa fluor 594 goat ant-mouse IgG, Invitrogen) was added and incubated at 37°C for another 40 min. Nonspecific staining as a control was performed by omitting primary antibodies.

### Statistical analysis

Significance levels were calculated using Student's t-test for unpaired samples. ρ<0.01 was regarded as statistically significant.
